# Keratin 15 protects against cigarette smoke-induced epithelial mesenchymal transformation by MMP-9

**DOI:** 10.1186/s12931-023-02598-w

**Published:** 2023-11-25

**Authors:** Wensi Zhu, Linxiao Han, Yuanyuan Wu, Lin Tong, Ludan He, Qin Wang, Yu Yan, Ting Pan, Jie Shen, Yuanlin Song, Yao Shen, Qiaoliang Zhu, Jian Zhou

**Affiliations:** 1 Department of Pulmonary and Critical Care Medicine, Shanghai Respiratory Research Institute, Zhongshan Hospital, Fudan University, 180 Fenglin Rd, Shanghai, 200032 China; 2 Department of Respiratory and Critical Care Medicine, Shanghai Pudong Hospital, 2800 Gongwei Rd, Shanghai, 201399 China; 3 Shanghai Institute of Infectious Disease and Biosecurity, Fudan University, Shanghai, 200032 China; 4 Shanghai Engineering Research Center of Internet of Things for Respiratory Medicine, 180 Fenglin Road, Shanghai, 200032 China; 5 Shanghai Key Laboratory of Lung Inflammation and Injury, 180 Fenglin Road, Shanghai, 200032 China; 6 Department of Thoracic Surgery, Zhongshan Hospital, Fudan University, 180 Fenglin Road, Shanghai, 200032 China; 7 Research Center for Chemical Injury, Emergency and Critical Medicine of Fudan University, Fudan University, Shanghai, 200540 China; 8 Center of Emergency and Critical Medicine in Jinshan Hospital of Fudan University, Fudan University, Shanghai, 200540 China; 9 Key Laboratory of Chemical Injury, Emergency and Critical Medicine of Shanghai Municipal Health Commission, Shanghai, 200540 China

**Keywords:** Airway remodeling, Chronic obstructive pulmonary disease, Inflammatory infiltration, Matrix metalloproteinase-9

## Abstract

**Background:**

Chronic obstructive pulmonary disease (COPD), a chronic inflammatory lung disease, is a leading cause of morbidity and mortality worldwide. Prolonged cigarette smoking (CS) that causes irreversible airway remodeling and significantly reduces lung function is a major risk factor for COPD. Keratin15^+^ (Krt15^+^) cells with the potential of self-renewal and differentiation properties have been implicated in the maintenance, proliferation, and differentiation of airway basal cells; however, the role of Krt15 in COPD is not clear.

**Methods:**

Krt15 knockout (Krt15^−/−^) and wild-type (WT) mice of C57BL/6 background were exposed to CS for six months to establish COPD models. *Krt15-CrePGR;Rosa26-LSL-tdTomato* mice were used to trace the fate of the Krt15^+^ cells. Hematoxylin and eosin (H&E) and Masson stainings were performed to assess histopathology and fibrosis, respectively. Furthermore, lentivirus-delivered short hairpin RNA (shRNA) was used to knock down KRT15 in human bronchial epithelial (HBE) cells stimulated with cigarette smoke extract (CSE). The protein expression was assessed using western blot, immunohistochemistry, and enzyme-linked immunosorbent assay.

**Results:**

Krt15^−/−^ CS mice developed severe inflammatory cell infiltration, airway remodeling, and emphysema. Moreover, Krt15 knockout aggravated CS-induced secretion of matrix metalloproteinase-9 (MMP-9) and epithelial–mesenchymal transformation (EMT), which was reversed by SB-3CT, an MMP-9 inhibitor. Consistent with this finding, KRT15 knockdown promoted MMP-9 expression and EMT progression in vitro. Furthermore, Krt15^+^ cells gradually increased in the bronchial epithelial cells and were transformed into alveolar type II (AT2) cells.

**Conclusion:**

Krt15 regulates the EMT process by promoting MMP-9 expression and protects the lung tissue from CS-induced injury, inflammatory infiltration, and apoptosis. Furthermore, Krt15^+^ cells transformed into AT2 cells to protect alveoli. These results suggest Krt15 as a potential therapeutic target for COPD*.*

**Supplementary Information:**

The online version contains supplementary material available at 10.1186/s12931-023-02598-w.

## Introduction

Chronic obstructive pulmonary disease (COPD) is the third most common cause of death and is associated with high morbidity and mortality rates worldwide. In 2019, approximately 212.3 million epidemic cases of COPD have been reported globally [1]. COPD is characterized by persistent airflow limitation and irreversible airway remodeling and can be diagnosed by the presence of a post-bronchodilator forced expiratory volume in one second/forced vital capacity value of < 0.7 (FEV1/FVC < 0.7) [2]. Smoking is the most common risk factor for COPD. Although the pathogenesis of COPD is still unclear, numerous studies have shown that long-term cigarette smoking (CS) exposure causes airway basal cell reprogramming and alveolar damage and triggers airway remodeling and emphysema (destruction and enlargement of alveoli) [3–5]. COPD increases the small airway resistance leading to obstruction of the air passage, which consequently causes obstructive bronchitis. Despite the high mortality rates [6] (3.3 million people die due to COPD) and the serious effects of COPD on human health and quality of life, a few drugs can repair damaged airways and promote lung regeneration.

The main pathological features of COPD include irregular inflammatory patterns and airway and parenchymal remodeling, which eventually leads to irreversible fibrosis and occlusion of small airways [7]. Epithelial–mesenchymal transition (EMT) plays an important role in small airway remodeling and subepithelial fibrosis. EMT is divided into three main types: type 1, which mainly occurs during embryonic development; type 3, which is significantly associated with epithelial malignancies; and type 2, which mainly occurs during organ fibrosis [8]. Several studies have shown that type 2 EMT is active in the small airways of patients with COPD [9–11]. During EMT, epithelial cells lose polarity and adhesion and then transit to a mesenchymal phenotype, which is characterized by a significant reduction in the expression of the epithelial cell marker E-cadherin and a significant increase in the expression of mesenchymal cell markers such as vimentin and S100A4 [12, 13]. At present, the drugs used for the treatment of COPD, such as bronchodilators, corticosteroids, and β2-agonists, can only alleviate the symptoms [14], but cannot reverse or delay airway remodeling. Therefore, further identification of the molecular mechanisms mediating EMT and the exploration of more effective and safe strategies that might attenuate EMT and hinder small airway remodeling is of immense practical benefit for preventing and treating COPD.

Keratin 5 (Krt5) dimerizes with Krt14 and forms the intermediate filaments underlying the basal epithelial cell cytoskeleton. Krt15, a type I keratin [15], has been shown to compensate for the lack of Krt14 and dimerizes with Krt5 to form keratin intermediate filaments [16]. Krt15 was first detected in the hair follicle bulges and its expression was shown to be correlated with epidermal cells [17]. Several studies have demonstrated that the expression of Krt15 is increased during hair follicle growth, differentiation, growth of the lacrimal gland, and epidermal injury repair [18–21]. In addition, Krt15^+^ precursor cells have been implicated in basal cell carcinoma [22, 23]. Krt15 has also been shown to be upregulated in a subset of urothelial cell carcinomas and mark the basal epithelia in ureters [24]. Krt15 also marks a population of long-lived progenitor cells in the mouse esophageal epithelium, which are capable of self-renewal, proliferation, and differentiation [25]. Krt15 has also been detected in the crypt cells of the small intestine, marking long-lived pluripotent crypt cells with self-renewal ability and radiation resistance, which play an important role in tissue regeneration after injury [20]. Together, these studies indicated that Krt15 is strongly associated with the repair and regeneration of tissue cells after injury. Recently, it has also been shown that when Krt15 in airway basal cells is switched to Krt14, the clonogenicity of airway basal cells decreases [26]. However, the role of Krt15 in COPD remains unclear.

We hypothesized that Krt15 mediates airway remodeling by regulating EMT. To validate this hypothesis, we first traced the dynamic changes in Krt15^+^ cells exposed to CS in reporter mice using genetic lineage tracing. The relationship between Krt15 and EMT was investigated by exposing Krt15^−/−^ mice to CS for 6 months and human bronchial epithelial (HBE) stimulated with cigarette smoke extract (CSE).

## Material and methods

### Bioinformatics and computational analysis

KRT15 mRNA expression data from RNA-seq datasets (GSE5058 and GSE8545) were obtained from the GEO database (https://www.ncbi.nlm.nih.gov/geo/). Only nonsmokers and patients with COPD were included in both datasets to analyze the expression levels of KRT15. The “GEOquery” [2.54.1] and “limma” [3.42.2] packages in R version 3.6.3 were used for the statistical analysis.

### Establishment of COPD models and animal grouping

C57BL/6 mice (8–10 weeks old) were purchased from Shanghai Model Organisms Center, Inc. (Shanghai, China). Krt15 knockout mice (Krt15^−/−^) were generated using the CRISPR-Cas9 gene editing technology at Shanghai Model Organisms Center, Inc. The mice were randomly divided into four groups based on their CS regimes: (I) wild-type (WT), (II) Krt15^−/−^, (III) WT CS, and (IV) Krt15^−/−^ CS with 6 mice in each group. WT CS and Krt15^−/−^ CS mice were exposed to CS for 6 months (4 h/day, 20 cigarettes/day, Daqianmen, Shanghai, China) in an acrylic box (75 × 75 × 45 cm) to establish COPD models [25, 27, 28].

*Krt15-CrePGR;Rosa26-LSL-tdTomato* mice were used to trace the fate of Krt15^+^ cells. These mice were intraperitoneally injected with 25 mg/kg/day RU486 (M8046, Sigma-Aldrich, Saint Louis, MO, USA) that were dissolved in corn oil for 5 days for Cre recombination. The mice were divided into six groups based on their smoking exposure: (I) R26^*mT*^-14d, (II) R26^*mT*^ CS-14d, (III) R26^*mT*^-30d, (IV) R26^*mT*^ CS-30d, (V) R26^*mT*^-60d, and (VI) R26^*mT*^ CS-60d groups, with three mice in each group. The R26^*mT*^ CS mice were exposed to CS for 14, 30, and 60 days.

Wild-type mice were intratracheally instilled with 60 mL adenovirus (5 × 10^10^ pfu/mL, Vigene Biosciences, Shangdong, China) to generate lung-specific Krt15 knockout (AdKrt15) mice. The mice were divided into (I) WT CS group, (II) AdKrt15 CS group, (III) AdKrt15 CS + SB-3CT group, and then were exposed to CS for 2 months. Mice in the AdKrt15 CS + SB-3CT group were intraperitoneally injected with 50 mg/kg SB-3CT which was dissolved in 10% Dimethyl sulfoxide (HY-Y0320, MCE), 20% Cremophor EL (HY-Y1890, MCE), and 70% ddH_2_O every other day for 14 days at 6 weeks after smoke exposure. All mice were housed in animal facilities at Zhongshan Hospital, Fudan University (Shanghai, China). All experimental procedures were approved by the Animal Care and Use Committee of Zhongshan Hospital, Fudan University (No.2022.032).

### Hematoxylin and eosin (H&E), Masson and TUNEL staining

The right upper lobes of lung tissues were infiltrated with 4% formaldehyde phosphate buffer for 24 h. The fixed lung tissue was subsequently embedded with paraffin and sectioned into 3 mm sections which were dewaxed by xylene three times and were soaked in 100%, 95%, and 75% alcohol in turn for further staining. For H&E staining, the sections were stained with hematoxylin dye and eosin dye. For masson staining, the sections were stained with hematoxylin for 5 min, then stained with ponceumic red for 10 min and phosphomolybdate for 5 min, and finally stained with aniline blue and differentiated with 1% acetic acid. For TUNEL staining, after the sections being repaired and the membranes being broken, TdT and dUTP were added at 2:29 and incubated at 37 °C for 2 h. Hydrogen peroxide solution (3%) was used to block endogenous peroxidase. After adding converter POD to each section, DAB was added and Harris hematoxylin was re-dyed.

### RNA-sequencing analysis

Total RNA was extracted from the lung tissue of the four groups (WT, WT CS, Krt15^−/−^ and Krt15^−/−^ CS) using TRIzol reagent (Thermo Fisher Scientific, Waltham, MA, USA) following the manufacturer’s protocol. Sequencing libraries were generated using NEBNext UltraTM RNA Library Prep Kit for Illumina (NEB, Houston, TX, USA). The clustering of the index-coded samples was performed on a cBot Cluster Generation System using TruSeq PE Cluster Kit v3-cBot-HS (Illumia, San Diego, CA, USA). RNA-sequencing analysis was performed by Shanghai Genechem Co., Ltd.(Shanghai, China).

### Immunofluorescence (IF)

Frozen sections of lung tissue were stained with rabbit anti-Sftpc antibody (1:500; DF6647, Affinity Biosciences, Cincinnati, OH, USA) overnight at 4 °C. Then the sections were incubated for 1 h at 37 °C with Alexa Fluor® 488 goat anti-mouse IgG (H+L) (A11001, ThermoFisher Scientific, Waltham, MA, USA). 4ʹ,6-diamidino-2-phenylindole (DAPI, C1006, Beyotime Biotechnology, Shanghai, China) was used to counterstain the nuclei.

### Immunohistochemistry (IHC)

After deparaffinization, the right lung lobes of four groups were incubated overnight at 4 °C with Krt15 (ab52816, Abcam), E-cadherin (3195T, Cell Signaling Technology, Boston, MA, USA), and Vimentin (5741T, Cell Signaling Technology) antibodies. The following day, sections were stained with horseradish peroxidase-conjugated secondary antibodies for 60 min at room temperature. 3,3ʹ-Diaminobenzidine staining was performed, and the results were observed under a microscope (Olympus, Tokyo, Japan).

### Enzyme-linked immunosorbent assay (ELISA)

The contents of MMP-9, IL-6, TNF-α, and IL-1β in BALF were detected using Mouse MMP9 DuoSet (DY6718, R&D systems, Minneapolis, MN, USA), IL-6 DuoSet (DY406, R&D systems), IL-1β DuoSet (DY401, R&D systems), and TNF-α DuoSet (DY410, R&D systems) ELISA kits, respectively, following the manufacture’s instructions. The MMP-9 content in the HBE cell supernatant was detected using a DuoSet ELISA kit (DY911-05, R&D Systems) according to the instructions supplied with the kit.

### Preparation of cigarette smoke extract (CSE)

Five cigarettes (Daqiammen, Shanghai Tobacco Group Co., Ltd. Shanghai, China) were lightly sucked into 10 mL serum-free cell culture media using a negative pressure aspirator at 6 min/mL. Subsequently, the CSE solution was filtered using 0.22 mm filters (SLGS033SS, Merck Millipore, MA, USA) and stored for further use. The optical density of the CSE (100%) at 320 nm is 4.1.

### Cell culture

HBE cells were purchased from the American Type Culture Collection and cultured in RPMI 1640 (Gibco, Carlsbad, CA, USA) containing 10% FBS (Gibco) and 1% penicillin–streptomycin (Gibco) at 37 °C in an incubator containing 5% CO_2_. For CSE exposure experiment, HBE cells were stimulated with 0%, 0.5%, and 1.5% CSE for 24 and 48 h.

### Lentiviral construction and infection

Lentivirus-containing short hairpin RNA (shRNA) targeting KRT15 and Lentivirus-vectors were pursued by Genechem (Shanghai, China). 5 × 10^4^ HBE cells were incubated in 6-well plates for 24 h in a 5% CO_2_ incubator. And the following day, the HBE cells were cultured in 1 mL medium with 5 mg/mL polybrene (Genechem) and a lentivirus-containing shRNA targeting KRT15 (shKRT15) or lentivirus-vectors (negative control). After incubating for 24 h, the medium was replaced with a fresh normal medium. These cells were divided into four groups based on their CSE and Lentivirus regimes: (I) Negative control (NC), (II) NC CSE, (III) shKRT15, (IV) shKRT15 CSE. The cells in NC CSE and shKRT15 CSE group were stimulated by 1.5% CSE for 48 h.

### Quantitative real-time-PCR (qRT-PCR)

RNA was extracted from lung tissue using RNA-Quick Purification Kit (RN001, Shanghai Yishan Biotechnology Co. Ltd., Shanghai, China) and was reverse-transcribed into cDNA using PrimeScript™RT reagent kit (RR037A, Takara Biotechnology, Osaka, Japan). qRT-PCR was performed to amplify the target genes (RR420A, Takara Biotechnology). The primer sequences are shown in Table 1.

**Table 1:** Primers used for quantitative real-time-PCR

Gene	Sequence(5ʹ → 3ʹ)
Mouse Krt15 forward	AGCTATTGCAGAGAAAAACCGT
Mouse Krt15 reverse	GGTCCGTCTCAGGTCTGTG
Human KRT15 forward	TCTGCTAGGTTTGTCTCTTCAGG
Human KRT15 reverse	CCAGGGCACGTACCTTGTC
Human MMP-9 forward	AGACCTGGGCAGATTCCAAAC
Human MMP-9 reverse	CGGCAAGTCTTCCGAGTAGT

*Krt15* Keratin 15, *MMP-9* Matrix metalloproteinase-9

### Cell viability assay

Approximately 1 × 10^4^ cells per well were seeded into 96-well plates and cultured for 24 h. At indicated time points (24 and 48 h), the medium in every well was replaced by new medium containing 10% CCK-8 solution (40203, Yeasen, Shanghai, China), and the plates were incubated for 1 h. Then the absorbance values at 460 nm were measured by FlexStation3 Multi-Mode Microplate Readers.

### Western blot analysis

The lung tissue was lysed by RIPA (P0013B, Beyotime Biotechnology, Shanghai, China) to obtain the protein. After the protein concentration of the right lung was measured using a spectrometer (NanoDrop 2000, Thermo Fisher Scientific, Waltham, MA, USA), the protein was first separated using sodium dodecyl-sulfate polyacrylamide gel electrophoresis, transformed into polyvinylidene difluoride membranes, and then blocked using QuickBlock™ Blocking Buffer (P0231, Beyotime Biotechnology). The polyvinylidene difluoride membranes were then stained with Krt15 (ab52816, Abcam), E-cadherin (3195T, Cell Signaling Technology), or Vimentin (5741T, Cell Signaling Technology) antibodies at 4 °C overnight. The following day, rabbit peroxidase-conjugated secondary antibodies (7074P2, Cell Signaling Technology) were used to detect target proteins.

### Statistical analysis

All data analyses were performed using GraphPad Prism9.0 software. A one-way analysis of variance was used for multiple group comparisons. All results are shown as the mean ± standard deviation (SD). Statistical significance was set at *p* < 0.05.

### KRT15 is overexpressed in patients with COPD and CS-exposed mice

**Fig. 1:**
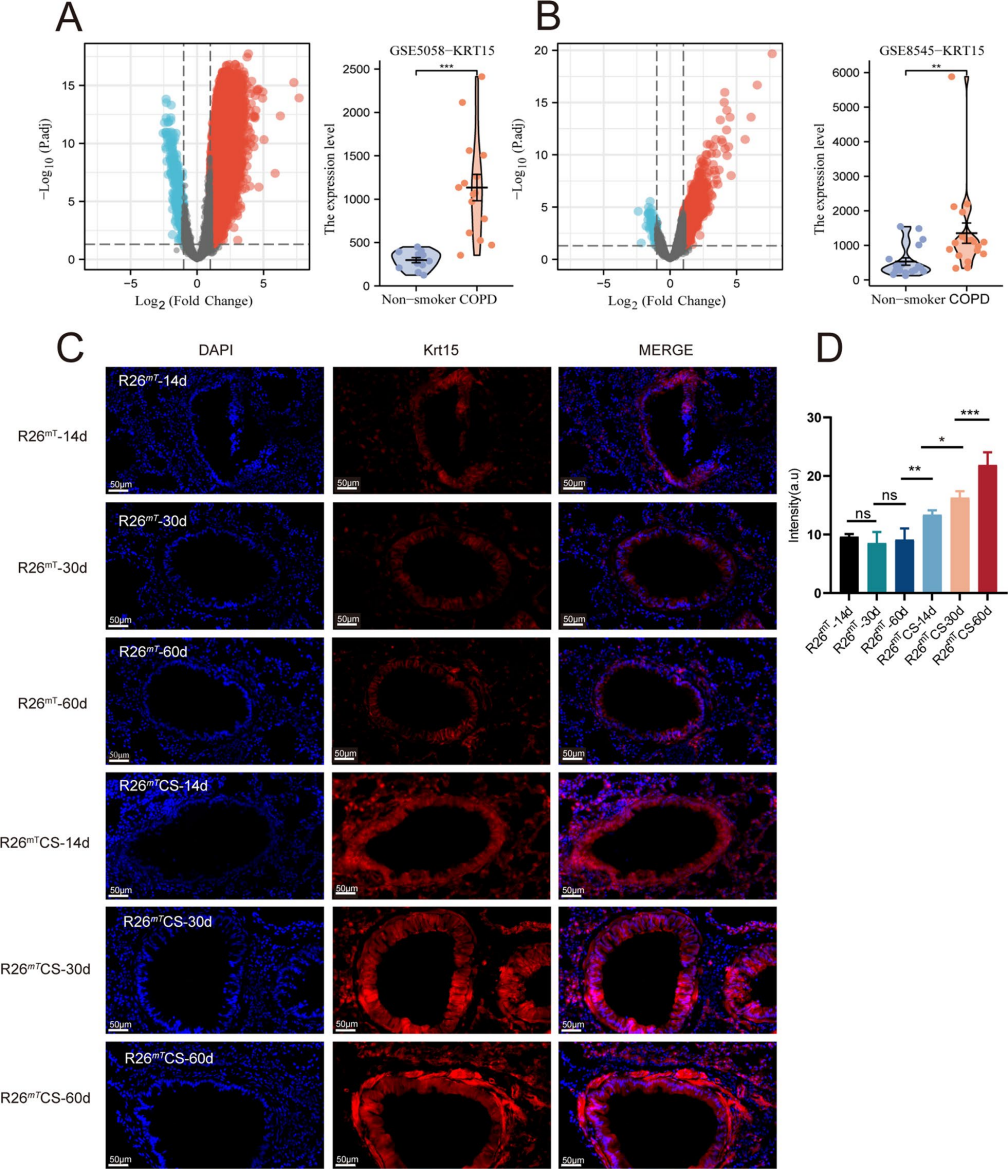
Expression of Krt15 in humans and mice. **A** and **B** Comparison of expression of Krt15 in nonsmokers and patients with COPD in GSE5058 and GSE8545 datasets obtained from the Gene Expression Omnibus database. **C** Immunofluorescence intensity of Tomato^+^ (Krt15^+^) cells (Red) in *Krt15-CrePGR;Rosa26-LSL-tdTomato* reporter mice; 4',6-diamidino-2-phenylindole (DAPI) was used to stain nuclei. **D** The intensity of Krt15^+^; N = 3 in each group.*p < 0.05; **p < 0.01; ***p < 0.001. R26^mT^-14d, R26^mT^-30d, R26^mT^-60d, *Krt15-CrePGR;Rosa26*-*LSL*-*tdTomato* mice exposed to air on days 14, 30, and 60; R26^mT^CS-14d, R26^mT^CS-30d, R26^mT^CS-60d, *Krt15-CrePGR*;*Rosa26*-*LSL*-*tdTomato* mice exposed to CS (on days 14, 30, and 60)

Estimation of the KRT15 expression in the two GEO datasets (GSE5058 and GSE8545) revealed a higher expression in patients with COPD (N = 33) than that in nonsmokers (N = 23) (*p* < 0.01; Fig. 1A and B).

To investigate the dynamic changes in Krt15^+^ cells following CS treatment in vivo, we used *Krt15-CrePGR;Rosa26-LSL-tdTomato* reporter mice to track Krt15^+^ cells [25, 27]. The intensity of Tomato^+^ (Krt15^+^) cells in the bronchial epithelial cells significantly increased in *Krt15-CrePGR;Rosa26-LSL-tdTomato* mice exposed to CS (on days 14, 30, and 60) compared to that in those not exposed to CS in exposure-duration dependent manner (Fig. 1C and D). Krt15^+^ cells in WT and WT CS groups did not show autofluorescence (Additional file 1: Figure S1).

### Knockout of Krt15 aggravates lung injury in CS-exposed mice

**Fig. 2:**
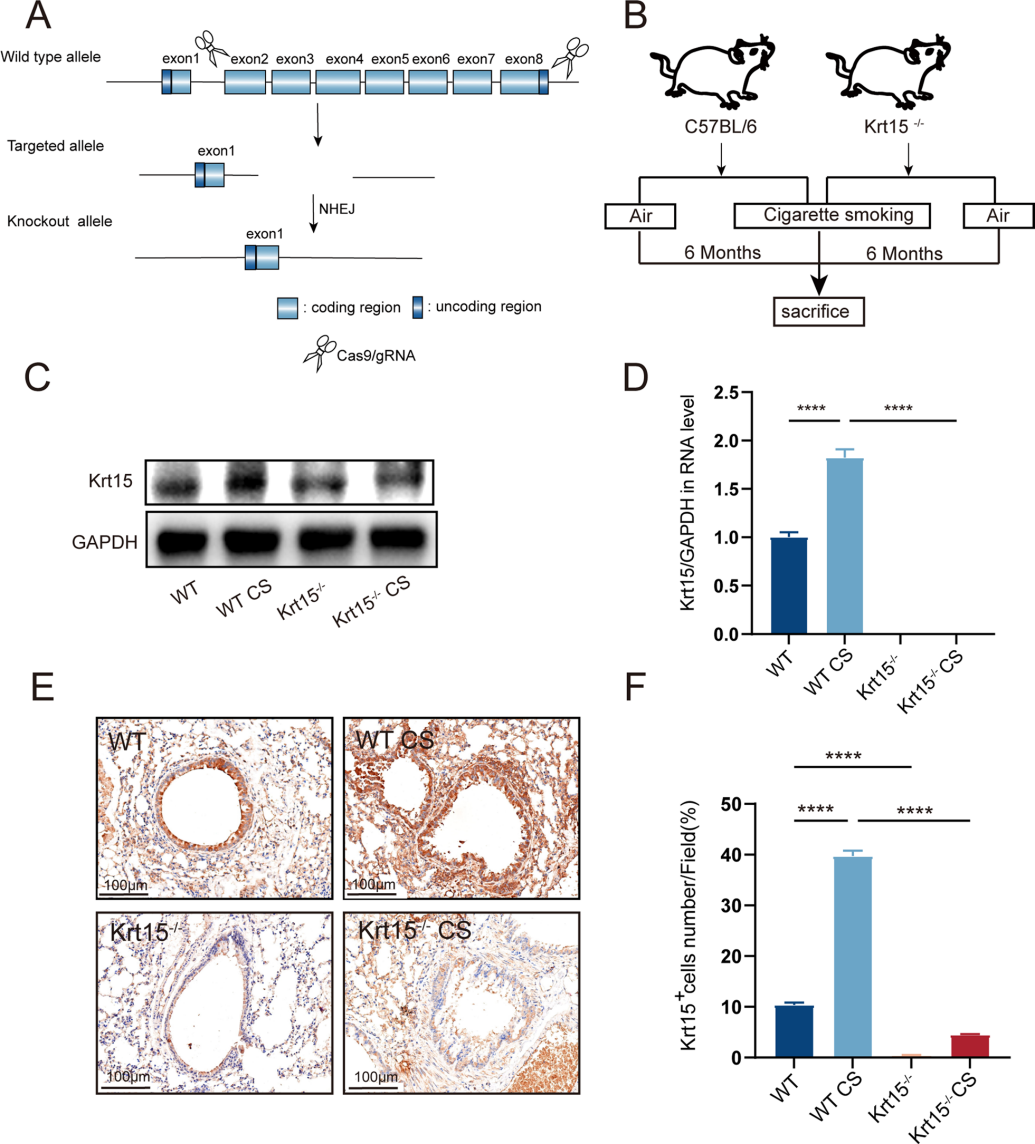
CRISPR/Cas9 gene editing technology to generate Krt15 knockout mice. **A** Schematic diagram of CRISPR/Cas9 gene editing to knockout *Krt15*. **B** Scheme of the establishment of mice COPD model by exposing WT CS and Krt15^−/−^ CS mice to cigarette smoking for six months, while the WT and Krt15^−/−^ mice were exposed to air for six months. **C**, **D** Expression of Krt15. **C** protein detected using western blot and **D** mRNA detected using qRT-PCR in the four groups; GAPDH was used as the internal control. **E** Immunohistochemistry (IHC) analysis of Krt15. N = 6 in each group. **F** Percentage of Krt15^+^ cells in IHC. ****p < 0.0001. *WT* wild-type, *WT CS* wild-type + CS exposure, *Krt15*^*−/−*^ Krt15 knockout mice, *Krt15*^*−/−*^* CS* Krt15 knockout mice + CS exposure, *CS* cigarette smoking

To investigate the role of Krt15 in COPD, the Krt15^−/−^ mice generated using the CRISPR-Cas9 (Fig. 2A) were exposed to CS for six months to establish a COPD model (Fig. 2B). The qRT-PCR and western blot results demonstrated that Krt15 mRNA and protein expression increased after CS exposure in the WT CS group compared to that in the WT group. In contrast, they were almost completely absent in the Krt15^−/−^ and Krt15^−/*−*^ CS groups (Fig. 2C and D). Consistent with this, IHC results showed that Krt15 expression increased after CS and decreased in the Krt15^−/−^ and Krt15^−/−^ CS groups (Fig. 2E and F).

**Fig. 3:**
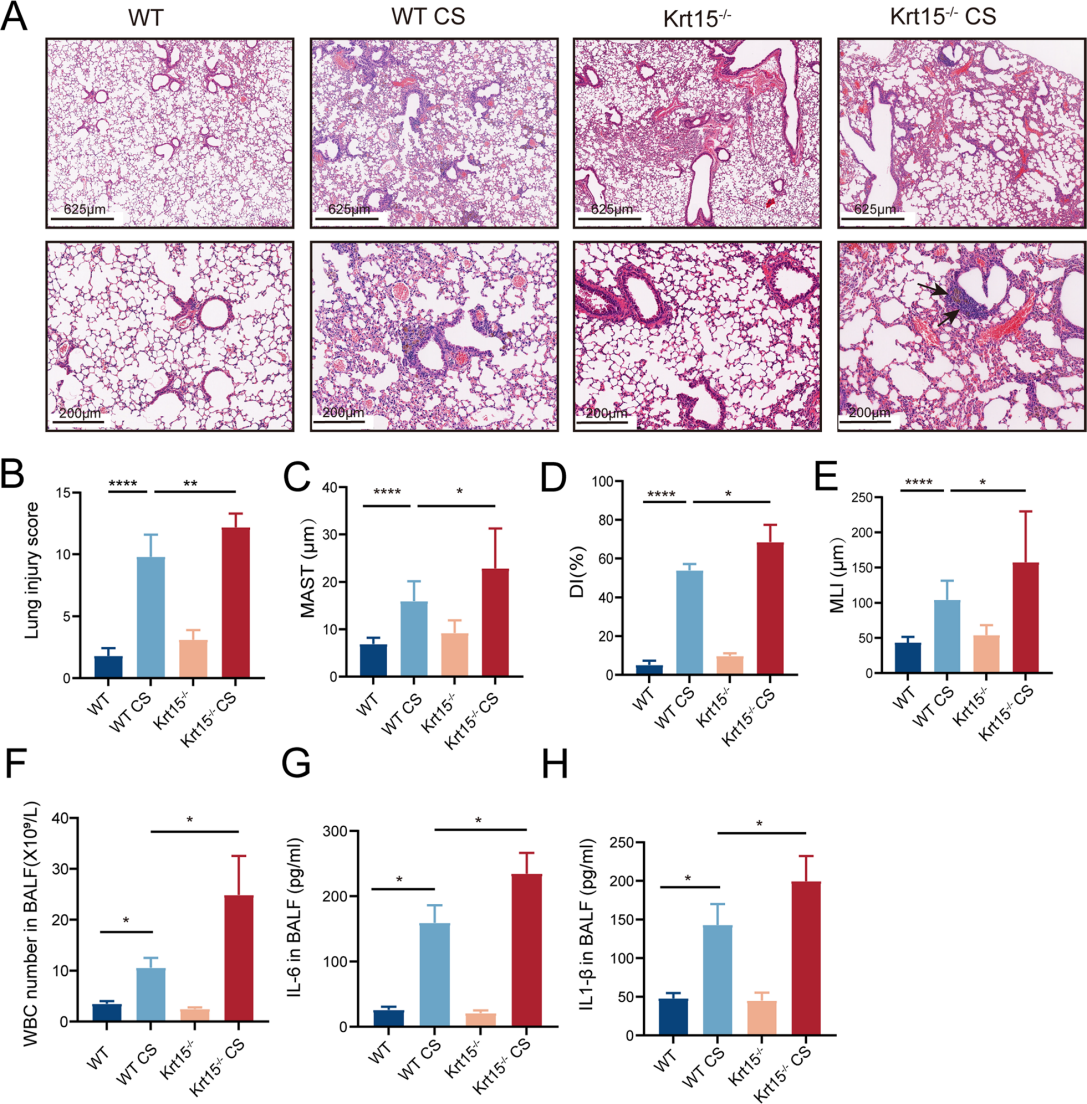
Knockout of Krt15 aggravates CS-induced lung injury and inflammatory infiltration. **A** H&E staining, **B** lung injury score, **C** MAST, **D** DI, **E** MLI, **F** WBC number in BALF, and **G** IL-6 and IL-1β (**H**) concentration. *p < 0.05; **p < 0.01; ***p < 0.001; ****p < 0.0001. *WT* wild-type, *WT CS* wild-type + CS exposure, *Krt15*^*−/−*^ Krt15 knockout mice, *Krt15*^*−/−*^* CS* Krt15 knockout mice + CS exposure, *CS* cigarette smoking. The arrows point to inducible bronchus-associated lymphoid tissue (iBALT)

H&E staining demonstrated more leukocyte infiltration, indicating higher inducible bronchus-associated lymphoid tissue (iBALT) in the WT CS group than that in the WT group, which was further increased in Krt15^−/−^ CS group (Fig. 3A). Subsequently, we evaluated the lung injury score [29], which revealed significantly higher lung injury scores in the Krt15^−/−^ CS group than those in the WT CS group (*p* < 0.01; Fig. 3B). Furthermore, the deficiency of Krt15 resulted in the thickening of the alveolar walls and airspace enlargement. The thickness of alveolar walls (mean alveolar septal thickness), percentage of destructive alveoli (destructive index), and mean linear intercept (MLI) [28, 30], were significantly increased in the WT CS group compared to those in the WT group, and further increased in the Krt15^−/−^ CS group (Fig. 3C–E). Moreover, the number of white blood cells and the secretion of IL-6 and IL-1β in BALF were significantly increased in the WT CS group compared to those in the WT group (*p* < 0.05 in all cases) and further increased in the Krt15^−/−^ CS group compared to those in the WT CS group (*p* < 0.05 in all cases) (Fig. 3F–H).

### Extracellular matrix plays a key role in Krt15-mediated regulation of COPD

**Fig. 4:**
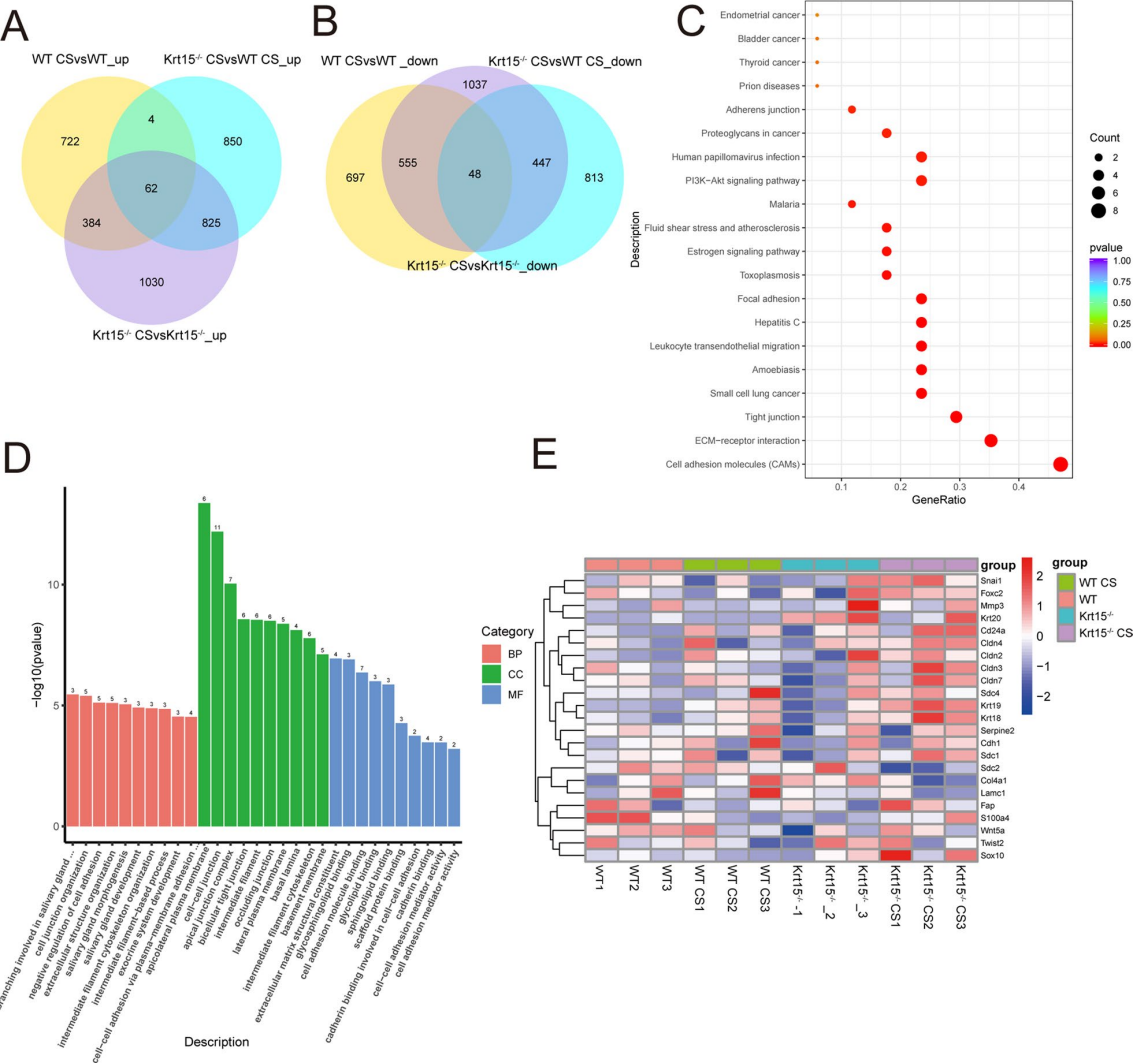
RNA-sequencing data analysis. **A** and **B** Venn diagrams of differentially expressed genes among WT, WT CS, Krt15^−/−^ and Krt15^−/−^ CS groups. **C** KEGG pathway analysis of differentially expressed genes. **D** GO analysis of differentially expressed genes. **E** Heatmap of top differentially expressed genes. N = 3 in each group

To investigate the specific mechanisms of Krt15 in the development and occurrence of COPD, we performed RNA sequencing. The Venn plot revealed that 1176 genes were upregulated in the WT CS group compared to those in the WT group. Among these genes, 66 genes were further upregulated in the Krt15^−/−^ CS group in comparison with WT CS group (Fig. 4A). Moreover, 1300 genes were downregulated in WT CS group compared to those in the WT group. Among these genes, 48 genes were further downregulated in the Krt15^−/−^ CS group in comparison with WT CS group (Fig. 4B). Kyoto Encyclopedia of Genes and Genomes (KEGG) analysis of these differentially expressed genes identified the enrichment of the cell adhesion molecules (CAMs) and ECM-receptor interaction pathways (Fig. 4C). Gene Ontology (GO) analysis showed that the genes were enriched in cell–cell junctions and extracellular matrix structural constituents (Fig. 4D). Figure 4F shows a heatmap of some of the top differentially expressed genes.

### Krt15 regulates the EMT progression by promoting MMP-9 expression

**Fig. 5:**
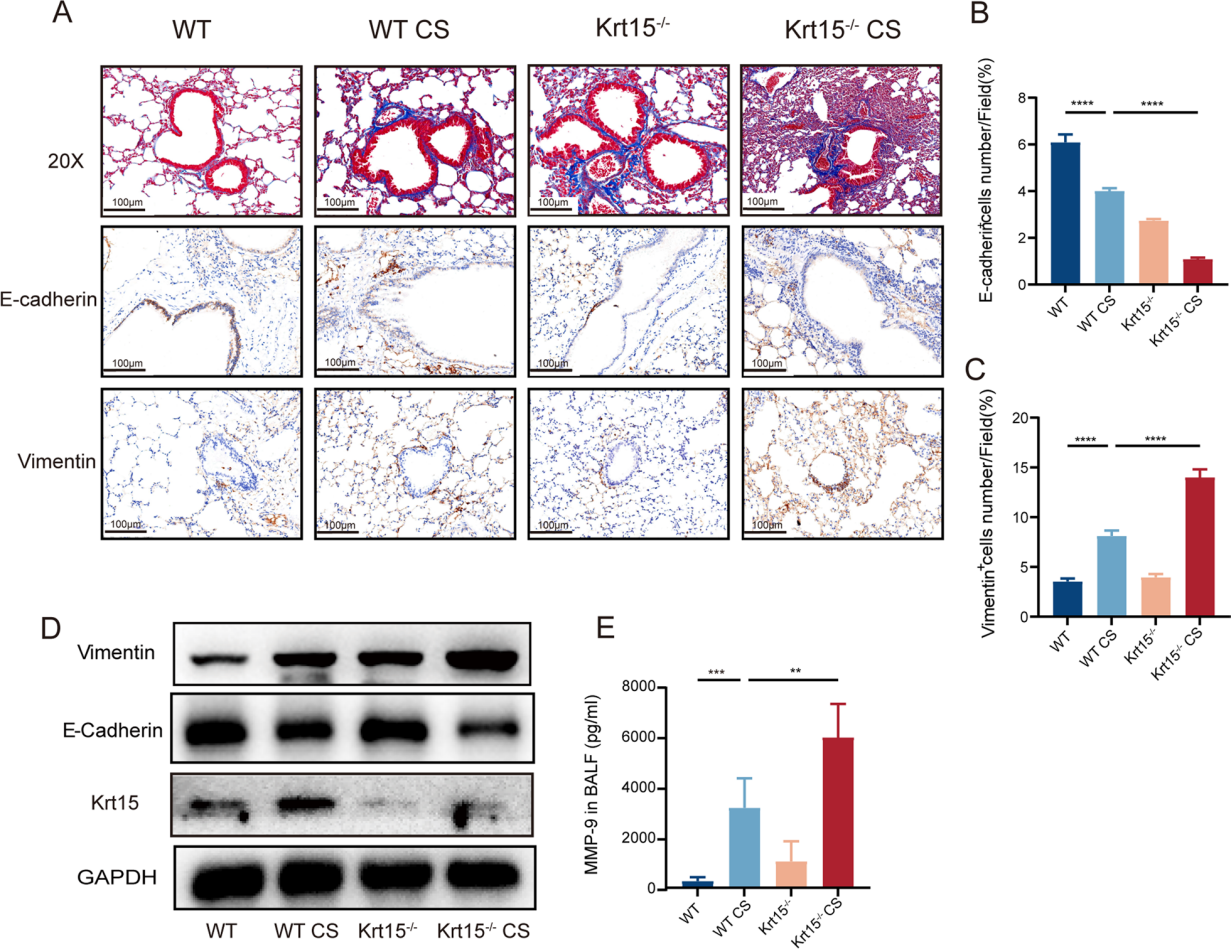
Krt15 regulates the EMT progression. **A** Masson staining and immunohistochemistry of E-cadherin and Vimentin. **B** and **C** expression of E-cadherin and Vimentin in IHC. **D** western blot analysis of Krt15, E-cadherin, and Vimentin. **E** MMP-9 concentration in BALF(pg/mL); **p < 0.01; ***p < 0.001

Based on the RNA sequencing analysis, we next explored the effect of Krt15 on EMT. Masson staining demonstrated that CS exposure for 6 months led to the development of a typical airway remodeling phenotype with significant collagen disposition and airway thickening in WT mice, which was further worsened in the absence of Krt15 (Fig. 5A). E-cadherin is often selected as a marker of the epithelium and Vimentin as a marker of the mesenchyme. IHC revealed that Krt15 knockout reduced the expression of E-cadherin and increased that of Vimentin in the Krt15^−/−^ CS group compared with those in the WT CS group (Fig. 5A–C). These results were consistent with the protein expression of E-cadherin and Vimentin measured using western blot (Fig. 5D).

**Fig. 6:**
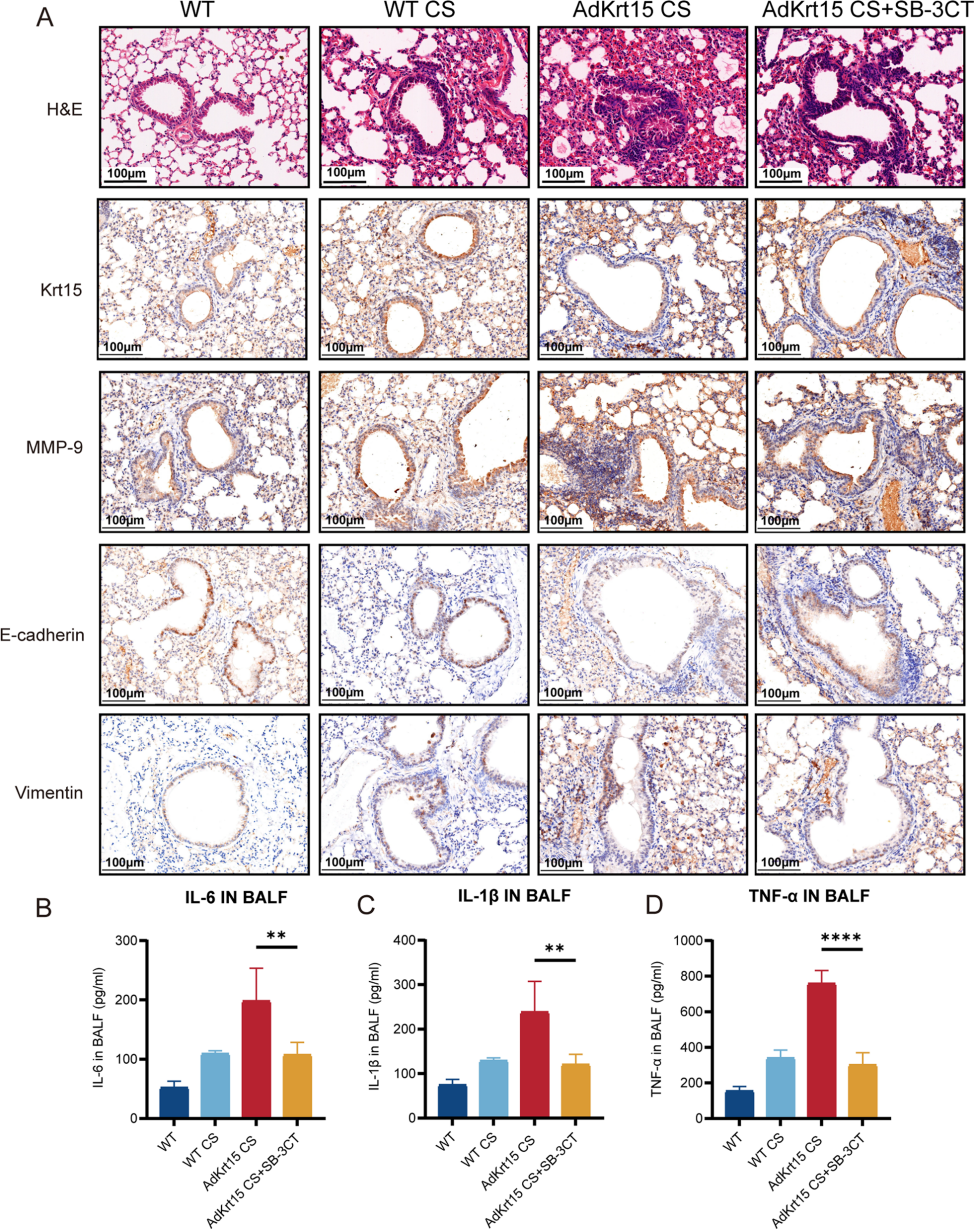
Inhibition of MMP9 alleviated the exacerbation of lung injury and inflammation caused by Krt15 knockdown in CS-exposed mice. **A** H&E and IHC stainings of Krt15, MMP-9, E-cadherin, and Vimentin. **B** The concentration of IL-6 (**B**); IL-1β (**C**); and TNF-α (**D**) in BALF. **p < 0.01, ****p < 0.0001. *WT* wild-type, *WT CS* wild-type mice + CS exposure, *AdKrt15 CS* wild-type mice + 60 mL adenovirus (5 × 10^10^ pfu/mL) + CS, *AdKrt15 CS + SB-3CT* wild-type mice + 60 mL adenovirus + CS + SB-3CT, *CS* cigarette smoking, *SB-3CT* an inhabitor of MMP-9

CS induces MMP-9 release, which has been shown to play a key role in EMT progression [31–33]. Therefore, we used ELISA to measure MMP-9 concentration in BALF. The results demonstrated that CS promoted MMP-9 expression, which was further increased by Krt15 knockout (Fig. 5E). To validate whether MMP9 mediates the role of Krt15 in the pathogenesis of COPD, we then generated lung-specific Krt15 knockout (AdKrt15) mice by intratracheally instilled with adenovirus. After intraperitoneal injection of SB-3CT, an MMP-9 inhibitor, for two weeks, it was found that mice in the AdKrt15 CS + SB-3CT group showed less airway remodeling and destruction of alveolar walls than those in the AdKrt15 CS group (Fig. 6A). Compared with AdKrt15 CS group, the expression of E-cadherin was increased while the expression of Vimentin was decreased in AdKrt15 CS + SB-3CT group (Fig. 6A). At the same time, SB-3CT could inhibit the release of inflammatory cytokines IL-6, TNF-α and IL-1β (Fig. 6B–D). The above results suggest that Krt15 regulates EMT progression by promoting MMP-9.

### KRT15 knockdown promotes MMP-9 expression and EMT progression in vitro

**Fig. 7:**
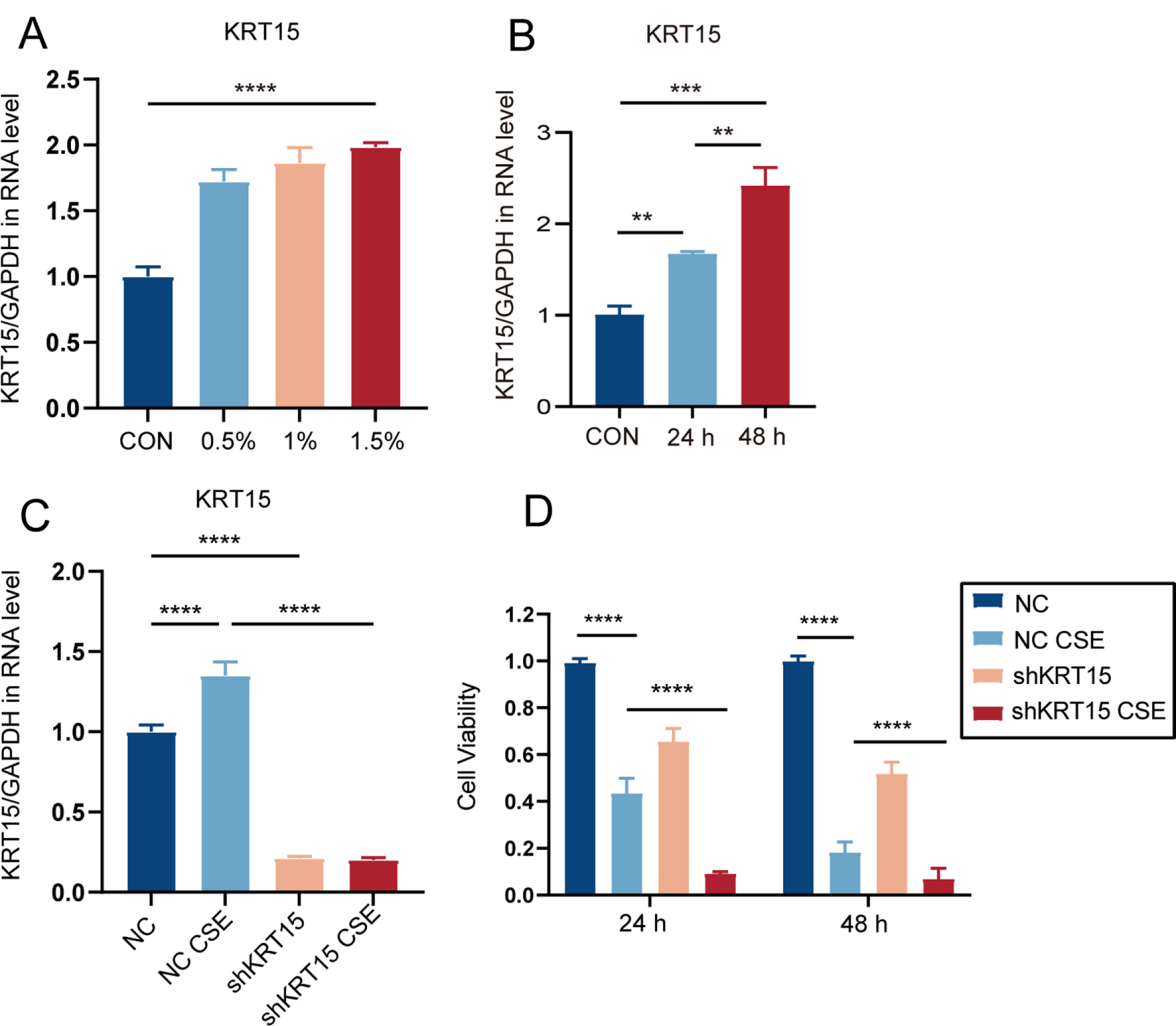
CSE stimulated Krt15 mRNA expression and reduced cell viability. **A** stimulated with 0.5%, 1%, and 1.5% CSE for 48 h, **B** with 1.5% CSE for 24 and 48 h, **C** treated with or without shRNA targeting KRT15 in the presence or absence of CSE. **D** Viability of HBE cells treated with or without shRNA targeting KRT15 in the presence or absence of CSE. ***p < 0.001;****p < 0.0001. *NC* negative control, *NC CSE* negative control + 1.5% CSE, *shKRT15* HBE cells using an shRNA targeting KRT15; shKRT15 CSE, HBE cells using an shRNA targeting KRT15 + 1.5%CSE

To confirm the role of KRT15 in vitro, HBE cells were stimulated with CSE to construct an in vitro COPD model. We first examined the mRNA expression of KRT15 at different time points and concentrations of CSE in HBE cells and found that CSE promoted the expression of KRT15 in a concentration- and time-dependent manner (Fig. 7A and B). HBE cells were exposed to 1.5% CSE for 48 h for subsequent experiments. Subsequently, we knocked down KRT15 in HBE cells using a shRNA targeting KRT15 (Fig. 7C). CCK-8 assay showed that the knockdown of KRT15 reduced cell viability in the shKRT15 CSE group compared to that in the NC CSE group at different time points (Fig. 7D).

**Fig. 8:**
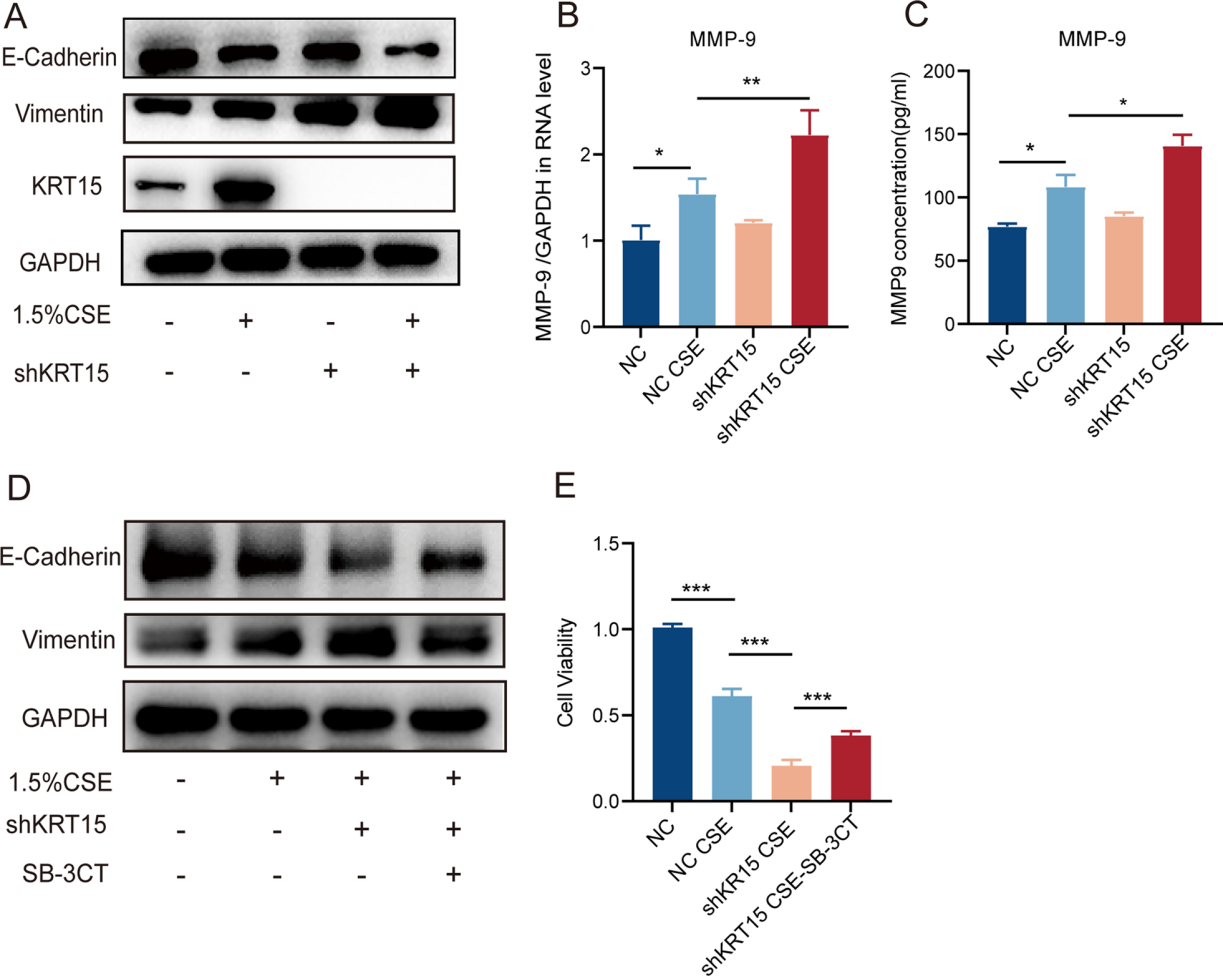
KRT15 knockdown promoted MMP-9 expression and EMT progression in vitro. **A** Western blot analysis of E-cadherin and Vimentin. **B** mRNA level of MMP-9 in HBE cells. **C** MMP-9 concentration in cellular supernatant. **D** Protein levels of E-cadherin and Vimentin. **E** Cell viability. *p < 0.05; **p < 0.01;***p < 0.001. *NC* negative control, *NC CSE* negative control + 1.5% CSE, *shKRT15* HBE cells using an shRNA targeting KRT15, *shKRT15 CSE* HBE cells using an shRNA targeting KRT15 + 1.5%CSE, *shKRT15 CSE-SB-3CT* HBE cells using an shRNA targeting KRT15 + 1.5%CSE + SB-3CT

Furthermore, western blotting to evaluate the EMT process in vitro revealed that KRT15 knockdown reduced the expression of E-cadherin and increased that of Vimentin in the shKRT15 CSE group compared to those in the NC CSE group (Fig. 8A). qRT-PCR and ELISA results demonstrated increased Mmp9 mRNA and protein expression in the NC CSE group compared to that in the NC group, whereas it was further increased in the shKRT15 CSE group compared to that in the NC CSE group in HBE cells (Figs. 8B and C).

To further confirm that Krt15 regulates EMT progression by promoting MMP-9, we treated HBE cells with SB-3CT for 2 h before CSE stimulation. SB-3CT reversed the reduction in E-cadherin and increase in Vimentin expression caused by the knockdown of KRT15 after CSE stimulation (Fig. 8D). Consistent with this, CCK-8 results showed increased cell viability in the presence of SB-3CT (shKRT15 CSE- SB-3CT group) compared to that in its absence (shKRT15 CSE group) (Fig. 8E).

### Krt15 protects the alveolar type II (AT2) cells during CS exposure

**Fig. 9:**
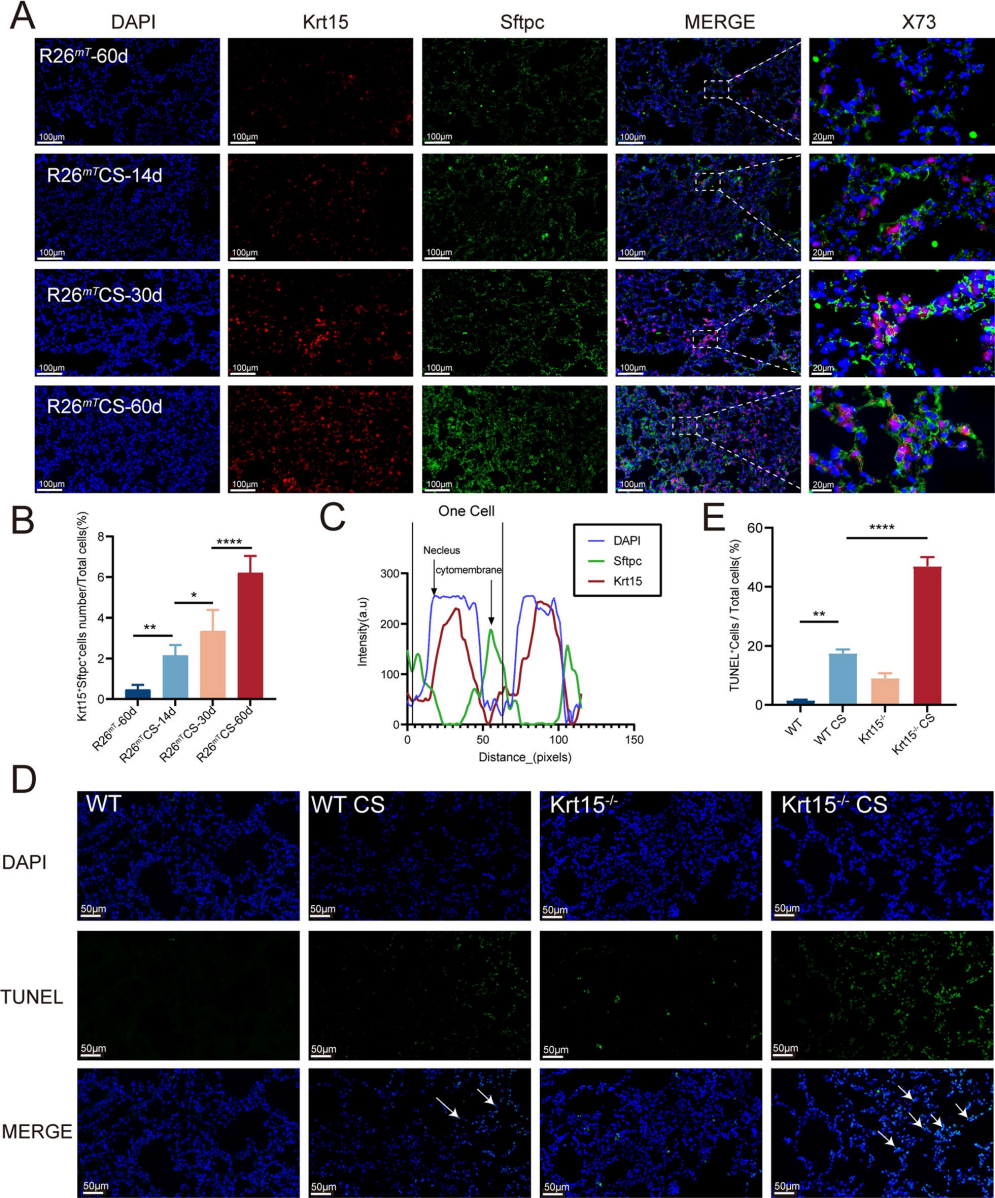
Krt15^+^ cells transform into AT2 cells to protect alveoli. **A** Immunofluorescence staining of lung tissue with Krt15 (Red) and Sftpc (Green). **B** The ratio of Krt15^+^Sftpc^+^ cells/Total cells. **C** Staining-intensity profiles showing signals from three fluorescent channels. **D** The ratio of TUNEL^+^ cells/Total cells. **E** TUNEL staining. N = 3 in each group.*p < 0.05; **p < 0.01; ***p < 0.001; ****p < 0.0001. R26^mT^-60d, *Krt1*5*-CrePGR*;*Rosa26-LSL-tdTomato* mice exposed to air on days 60; R26^mT^CS-14d, R26^mT^CS-30d R26^mT^CS-60d, *Krt15-CrePGR; Rosa26*-*LSL*-*tdTomato* mice exposed to CS (on days 14, 30, and 60). *WT* wild-type, *WT CS* wild-type + CS exposure, *Krt15*^*−/−*^ Krt15 knockout mice, *Krt15*^*−/−*^* CS* Krt15 knockout mice + CS exposure, *CS* cigarette smoking. The arrows point to TUNEL^+^ cells

H&E staining results demonstrated that CS exposure induced serious destruction and enlargement of alveoli in Krt15^−/−^ mice (Fig. 3A). Furthermore, genetic lineage tracing revealed increased Tomato^+^ (Krt15^+^) cells in the alveoli after CS exposure (Fig. 9A). To test whether Krt15^+^ cells transform into AT2 cells, we performed immunofluorescence experiments to double-stain Krt15 and Sftpc, a marker of AT2 cells. CS exposure increased the number of Tomato^+^ (Krt15^+^)/Sftpc^+^ cells in *Krt15-CrePGR; Rosa26-LSL-tdTomato* mice on days 14, 30, and 60 compared to that without CS exposure on day 60 (Fig. 9B and C). Furthermore, the TUNEL staining assessment showed that there were more apoptotic cells in the alveoli of Krt15^−/−^ CS group than those of WT CS group (Fig. 9D and E).

## Discussion

COPD is an irreversible disease characterized by airway remodeling and accelerated decline in lung function [34–36], which significantly affects the quality of life in patients and increases economic and social burden worldwide [6]. Although it is one of the leading causes of death, the existing treatment methods are limited and can mainly alleviate the clinical symptoms of COPD. None of the current treatment methods are efficient in improving lung function and mortality in patients with COPD [5, 37, 38]. Therefore, identifying the endogenous regeneration mechanisms that can restore the decline in lung function is important to treat COPD [39]. KRT15 identified as a stem cell marker in hair follicles [40], has gained recognition in identifying long-lived progenitor cells in the esophageal epithelium and small intestine crypts [20, 25]. Moreover, a recent study has demonstrated its potential role in airway basal cells to compensate for the lack of Krt14 that forms the intermediate filaments for establishing the basal epithelial cytoskeleton [26]. In this study, we explored the potential role of Krt15 in COPD and its underlying molecular mechanisms.

Our findings revealed a significantly higher expression of KRT15 in patients with COPD than that in nonsmokers. We also showed for the first time that Krt15^+^ cells were mainly expressed in the bronchial epithelium, and their expression increased with an increase in CS exposure time. In addition, both mRNA and protein expression of Krt15 in the lung tissues of mice significantly increased after exposure to CS. In addition, after stimulating HBE cells with CSE, KRT15 expression significantly increased in a time- and concentration-dependent manner. These results suggest that Krt15 plays an important role in the occurrence and development of COPD.

Furthermore, CS exposure for six months accelerated the severity of lung injury in Krt15^−/−^ mice and more significant tertiary lymphoid structure formation was observed in iBALT [41]. iBALT can cause the recruitment of inflammatory cells and the destruction of alveolar walls that is related to emphysema during chronic CS exposure [38, 42–44]. In consistent with this, the highest percentage of alveolar destruction and secretion of inflammatory cytokines IL-6 and IL-1β was in the Krt15^−/−^ CS group (Fig. 3G and H).

Next, we assessed the degree of emphysema by measuring MLI in the four groups [45]. After exposure to CS for six months, the MLI in the Krt15^−/−^ CS group was higher than that in the WT CS group. In the Krt15^+^ cell-tracing experiment, the number of Tomato^+^(Krt15^+^)/Sftpc^+^ cells increased significantly with increased CS exposure time. Sftpc is often used as a marker for AT2 cells [46, 47], which act as stem cells during lung injury [48]. Prolonged exposure to CS disposes of AT2 cells to senesce and apoptosis [49, 50], consequently leading to failure in alveolar wall maintenance, which results in emphysema and hinders the damage repair [51]. Previous studies have demonstrated that the stemness of AT2 cells increases to defend against CS-induced lung injury, but fail to conpensate the damage of AT2 cells and avoid emphysema [52]. Furthermore, TUNEL staining identified an increased number of apoptotic cells in the alveolar region in the Krt15^−/−^ CS group compared to the WT CS group in this study. These results indicate that Krt15 plays a vital role in protecting alveoli.

Conversely, the results of RNA-sequencing analysis suggest that the role of Krt15 in COPD is related to the structural constituents of the extracellular matrix. Both IHC and western blot results demonstrated that CS exposure decreased the expression of E-cadherin and increased that of Vimentin in Krt15 knockout mice. Because E-cadherin and Vimentin play key roles in EMT [9], we inferred that Krt15 knockout leads to increased airway remodeling in mice by promoting EMT progression. The in vitro experiments confirmed these in vivo results. After CSE stimulation, shKRT15 cells exhibited higher Vimentin expression, lower E-cadherin expression, and decreased cell viability.

CSE and CS can stimulate the expression of MMP-9 [31, 53, 54], and multiple studies have shown that MMP-9 is highly correlated with EMT processes. Recombinant MMP-9 induces EMT in the lung epithelium and glomerular endothelium [32, 55]. We demonstrated that Krt15^−/−^ mice and shKRT15 cells secreted more MMP-9 upon stimulation with CSE and CS, respectively. Furthermore, SB-3CT reversed the CSE-induced alterations in E-cadherin and Vimentin expression and increased cell viability. These results prove that silencing of Krt15 promotes EMT by upregulating the expression of MMP-9.

## Conclusion

Here, we established the mice models of COPD and unraveled the potential role of Krt15 in COPD and its underlying molecular mechanisms. The findings of the present study demonstrate that Krt15 negatively regulates EMT by promoting MMP-9 expression and protecting alveoli structure. Overall, these findings suggest Krt15 is an important target for COPD treatment.

### Supplementary Information

**Additional file 1: Figure S1.** Krt15^+^ cells did not show autofluorescence. WT, wild-type; WT CS, wild-type + CS exposure of 60 days.

## Data Availability

Not applicable.

## Abbreviations

Krt15: Keratin15
COPD: Chronic obstructive pulmonary disease
CS: Cigarette smoking
WT: Wild-type
HBE: Human bronchial epithelial
CSE: Cigarette smoke extract
MMP-9: Matrix metalloproteinase-9
EMT: Epithelial–mesenchymal transformation
iBALT: Inducible bronchus-associated lymphoid tissue
