# Supplementary material for: Keratin 15 protects against cigarette smoke-induced epithelial mesenchymal transformation by MMP-9
Source: Respir Res. 2023 Nov 25;24:297. doi: 10.1186/s12931-023-02598-w (PMC10675954; doi:10.1186/s12931-023-02598-w)
Supplement: Supplementary file 1 — Additional file 1: Figure S1. Krt15+ cells did not show autofluorescence. WT, wild-type; WT CS, wild-type + CS exposure of 60 days. [file 12931_2023_2598_MOESM1_ESM.pdf]

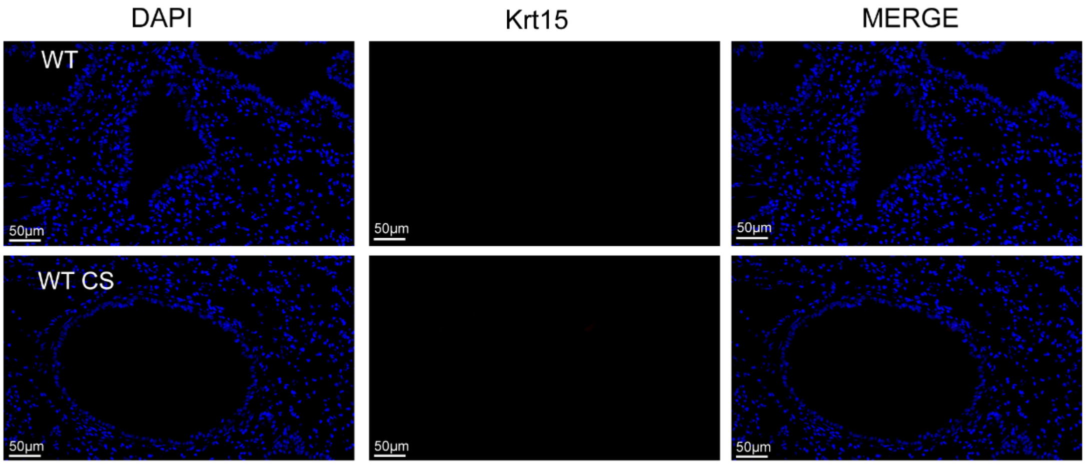

Figure S1: Krt15^{+} cells did not show autofluorescence. WT, wild-type; WT CS, wild-type + CS exposure of 60 days.
